# Characterization of a Temperature-Sensitive Vertebrate Clathrin Heavy Chain Mutant as a Tool to Study Clathrin-Dependent Events *In Vivo*

**DOI:** 10.1371/journal.pone.0012017

**Published:** 2010-08-06

**Authors:** Petra Neumann-Staubitz, Stephanie L. Hall, Joseph Kuo, Antony P. Jackson

**Affiliations:** 1 Department of Biochemistry, University of Cambridge, Cambridge, United Kingdom; 2 Institut for Microbiology and Genetics, Department for General Microbiology, Georg-August-University of Goettingen, Goettingen, Germany; University of Geneva, Switzerland

## Abstract

Clathrin and clathrin-dependent events are evolutionary conserved although it is believed that there are differences in the requirement for clathrin in yeast and higher vertebrates. Clathrin is a long-lived protein and thus, with clathrin knockdowns only long-term consequences of clathrin depletion can be studied. Here, we characterize the first vertebrate temperature-sensitive clathrin heavy chain mutant as a tool to investigate responses to rapid clathrin inactivation in higher eukaryotes. Although we created this mutant using a clathrin cryo-electron microscopy model and a yeast temperature-sensitive mutant as a guide, the resulting temperature-sensitive clathrin showed an altered phenotype compared to the corresponding yeast temperature-sensitive clathrin. First, it seemed to form stable triskelions at the non-permissive temperature although endocytosis was impaired under these conditions. Secondly, as a likely consequence of the stable triskelions at the non-permissive temperature, clathrin also localized correctly to its target membranes. Thirdly, we did not observe missorting of the lysosomal enzyme beta-glucuronidase which could indicate that the temperature-sensitive clathrin is still operating at the non-permissive temperature at the Golgi or, that, like in yeast, more than one TGN trafficking pathway exists. Fourthly, in contrast to yeast, actin does not appear to actively compensate in general endocytosis. Thus, there seem to be differences between vertebrates and yeast which can be studied in further detail with this newly created tool.

## Introduction

Eukaryotic cells are in constant exchange with their environment and steadily adapt to it by modulating their plasma membrane composition. Continuous cargo flow between the plasma membrane and intracellular compartments is achieved by intracellular vesicle trafficking. Vesicle formation on donor compartments often depends on special coat proteins such as clathrin serving as cage scaffolds [1]. Clathrin-dependent vesicle budding starts with the assembly of clathrin with its adaptor and accessory proteins at the donor membrane into curved basket-like lattices which cause the deformation of the membrane patch and transform it into a vesicle (clathrin coated vesicles, CCV). In this process selected membrane proteins are sequestered into the vesicle. After pinching-off, the clathrin coat is rapidly disassembled to allow the vesicle to fuse with the next compartment. Clathrin coated vesicles serve numerous crucial functions within a cell: Clathrin chiefly controls the major receptor-mediated endocytosis pathway of selected receptors at the plasma membrane [2], [3] and is also responsible for selective protein sorting at the *trans*-Golgi network (TGN) for transport to lysosomes and secretory granules [4]. In synapses, CCVs are involved in the recycling of synaptic vesicles and the uptake of neurotransmitters (for review see [5] and [6]). Interestingly, as an additional function to vesicle formation, clathrin has been implicated in the control of mitosis by stabilizing the mitotic spindle fibres when no membrane trafficking occurs [7], [8], [9].

Clathrin is a highly conserved, soluble, stretched protein consisting of three heavy (∼190 kDa) and three associated light chains (∼25 kDa). The C-termini of heavy and light-chain complexes trimerize with each other in the “hub” region to form a stable three-legged structure called triskelion. The controlled assembly and disassembly of triskelions dictates the formation of the clathrin-coated vesicles (CCVs) [5].

The role of clathrin *in vivo* has been investigated substantially by inactivating clathrin. A number of knockouts and knockdowns in various cell types have been performed (for review see [6]) for example by creating a dominant negative clathrin mutant [10] or by siRNA-approaches [8], [11], [12], [13], [14]. In yeast two independent clathrin knockout mutants have been generated by homologous recombination [15], [16]. This method is not easily adaptable to higher eukaryotic cells, since these lack an efficient homologuous recombinatory machinery required for genetic manipulation purposes. However, one remarkable exception is the chicken pre-B-lymphocyte cell line DT40 [17], [18], [19]. Here, Wettey et al. achieved a conditional clathrin heavy chain knockout where both endogenous chicken clathrin heavy chain alleles were disrupted and a 96% identical human cDNA clathrin heavy chain under a inducible promoter (Tet-off system) [17] was introduced. Cells grown in the presence of doxycycline showed a very tight repression of clathrin expression. The original clathrin-regulatable cell line underwent apoptosis in the absence of clathrin, however, a variant cell line survived under defined growth conditions. As expected, clathrin-repression in these clathrin knockout cell lines significantly reduced receptor-mediated endocytosis. Surprisingly however, the targeting of lysosomal proteins was unaffected [17] which is in agreement with the yeast clathrin heavy chain knockout [16], [20]. Yet, clathrin is a very stable protein which can take days [17], [21] to get degraded and so, with knockdown and knockout systems it is not possible to discriminate between short-term or long-term clathrin depletion effects. Generally, consequences of short-term inactivation of an essential protein can be investigated by a mutated variant of this protein, which carries out its function at permissive conditions whereas it is blocked at non-permissive conditions.

Three temperature sensitive-clathrin heavy chain mutants (TS-clathrin) have been created in yeast, and another in *C. elegans* [22], [23], [24], [25]. All mutations are genetically different but are located within or close to the trimerization domain. All yeast TS-clathrin mutants [22], [23], [24] predominantly addressed clathrin-dependent sorting at the TGN, whereas the authors of the *C. elegans* TS-mutant were chiefly interested in synaptic vesicle pool maintenance, where clathrin seems to be dispensable [25]. Yeast TS-clathrin mutants showed impaired endocytosis and a defect in TGN-trafficking at the non-permissive temperature concerning secretion of alpha-factor (mating pheromone) and sorting of the vacuolar protein carboxypeptidase Y (CPY) whereas the vacuolar protein alkaline phosphates (ALP) was unaffected [22], [23], [24], [26]. Thus, there are at least two independent TGN lysosomal trafficking pathways in yeast, one clathrin dependent (CPY-pathway), one independent (ALP-pathway) (for review see [27]).

Clathrin itself and clathrin-mediated vesicle formation are highly conserved in evolution and one could speculate that the major protein homologues involved in CCV-formation have similar importance in different species. Hence, different cell-types would behave similarly if one of the main proteins is rapidly inactivated. However there is an ongoing debate as to whether there are significant differences in the requirement for clathrin and actin in endocytosis. The main reasons for this discussion are firstly the observation that some yeast clathrin knockout and knockdown strains survive even though they show severe defects for example in growth [16], [28], [29] and secondly, effects of actin inhibition on endocytosis in higher eukaryotes are described as partial [30], [31], [32], [33], [34], [35]. It is proposed that yeast endocytosis relies more on actin than on clathrin whereas in mammalian cells this is vice versa (for review see [30], [31], [32], [33], [34]). In a yeast TS-clathrin mutant, missorting of TGN-derived vesicles was observed after a shift to the non-permissive conditions which reverted to normal after prolonged incubation [22]. This is also consistent with the clathrin knockout in DT40 where no missorting of Golgi-derived vesicles could be observed after long-term clathrin-depletion for more than three days, suggesting an adaptation by up-regulating compensatory mechanisms. Due to the sorting-defects in rapidly inactivated clathrin in yeast and the indications for differences between yeast and mammalian cells in the evolutionary highly conserved clathrin coated vesicle formation process, we became interested in how vertebrate cells would respond to short-term clathrin inactivation. As a tool we generated the first temperature-sensitive mutant of a vertebrate clathrin, in the chicken pre-B cell line DT40, based on one of the TS-mutants in yeast [36] and a cryo-EM model of bovine clathrin [37].

## Results

### A temperature-sensitive vertebrate clathrin heavy chain mutant

**Figure 1:**
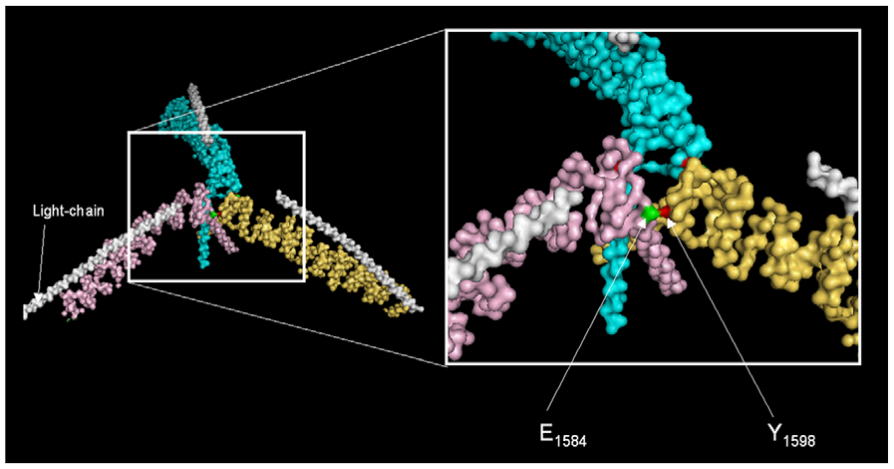
Creating a temperature-sensitive vertebrate clathrin heavy chain. The image shows the cryo-electron microscope graph of assembled bovine clathrin [37]. The three heavy chains (coloured in light blue, pink and yellow) trimerize in a region called “hub”. The associated clathrin light chains are shown in grey. The augmented image shows the two amino acid residues, E1584 (green) and Y1598 (red) of the neighbour clathrin heavy chain, which make contact with each other according to the model. We genetically manipulated the E1584 residue to a lysine (E1584K) according to the yeast TS-clathrin mutant [22], [38].

Clathrin is a long-lived protein with a half life of 24–36 hours [21]. Yet, it takes four days following doxycycline treatment for all detectable clathrin to completely disappear in a DT40 cell line containing the human clathrin heavy chain gene (cDNA) under a doxycycline controllable promoter (as the only source of clathrin) (DKO-S cell line; conditional clathrin knockout cells, undergoing apoptosis in absence of clathrin; see table 1) [17]. Thus, we expected that some of the functional effects of clathrin removal may be abrogated by compensatory changes during this time period and that these would obscure or complicate experimental analysis. To investigate the effect of short-term clathrin inactivation we set out to generate a stable DT40 cell line that constitutively expresses a temperature-sensitive (TS) clathrin allele. Several TS-clathrin heavy chain mutants have been generated in yeast and *C. elegans* and all were mapped to the trimerization domain. One of these TS-clathrin mutants was a frameshift mutant [24] two were deletion mutants [23], [25] and one carried several point mutations [22], [38] but further analysis showed that glutamic acid (E1590) mutated to lysine within the trimerization region (hub) was the most important one for the TS-phenotype [36]. This glutamic acid residue and its surrounding sequence have been conserved during eukaryotic evolution, suggesting a functional importance. The importance of the amino acid residue E1590 in yeast or its human homolog E1584 is underlined by and consistent with the cryo-EM structure of clathrin [37] (figure 1). The model suggests that the glutamic acid residue lies close to or within the trimerization domain of clathrin heavy chain [37] interacting with a tyrosine (Y1598) residue of the neighbouring heavy chain. As deletions or truncations of a protein can interfere with protein folding and can impact its structure, we decided to mutate this one important residue (E1584) to minimize any disturbance. Human clathrin heavy chain cDNA is 96% identical to chicken clathrin heavy chain and has previously been used to rescue a clathrin knockout DT40 cell line [17]. We therefore used it to convert residue E1584 to lysine (E1584K) using site-directed mutagenesis (figure 1).

**Table 1:** Cell lines used in this study.

Abbreviation used in text	Information about clathrin heavy chain expression	reference
DKO-S (original wild type)	Grown without doxycycline, DKO-S cells express human cDNA clathrin heavy chain under a Tet-off promoter (original wild-type)	[17]
clathrin *knockout*	DKO-S cells grown with doxycycline cause a repression of humancDNA clathrin expressed via the Tet-off promoter; now clathrin heavy chain gets degraded within several days	[17]
wild type	DKO-S cells with inserted human cDNA clathrin heavy chain under a CMV-promoter: doxycycline causes repression of clathrin expression under the Tet-off promoter, whereas clathrin heavy chain is constitutively expressed via the CMV promoter	this study
TS-mutant	DKO-S cells with inserted human cDNA clathrin heavy chain with E1584K TS-mutation under a CMV promoter: doxycycline causes repression of clathrin expression under the Tet-off promoter whereas TS-clathrin heavy chain is constitutively expressed via the CMV promoter (TS26, TS-mutant)	this study

To generate and select for a stable DT40 cell line capable of expressing TS-clathrin, DKO-S cells were transfected with a plasmid encoding the full-length human clathrin E1584K mutant (cDNA) under a strong and constitutive CMV promoter. These cells were grown in media with doxycycline (to completely repress wild-type clathrin expression) and only 0.3% chicken serum. It has been shown previously that cells without clathrin die under low chicken serum supplementation [17]. Additionally, cells were grown at 30°C to minimise any TS-effect at this stage. Under these conditions, non-transfected cells died after 7–10 days due to a complete loss of clathrin [17], but stably transfected clones survived. We tested the surviving clones for their ability to grow at 30°C as opposed to 42°C and confirmed the disappearance or presence of clathrin heavy chains by immuno fluorescence stain. A total of 26 clones were isolated all showing a TS-phenotype. These cell lines can therefore be grown in presence of doxycycline to repress their wild-type clathrin so that only the TS-mutant clathrin heavy chain is expressed. For consistency, all further work was done with one E1584K clone (TS26) designated as TS-mutant.

### Growth at permissive and non-permissive temperature

**Figure 2:**
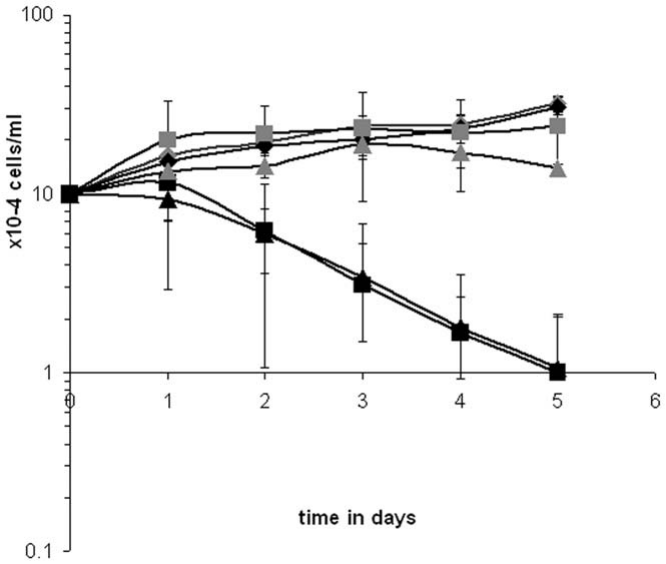
Growth at permissive and non-permissive temperature. Prior to the growth experiment, indicated cell lines were grown for four days with doxycycline to repress clathrin expressed via the Tet-off promoter. For the assay cells were diluted to 1×10^5^ cells/ml into fresh medium and were incubated at 30°C (permissive temperature) or 42°C (non-permissive temperature) in presence of doxycycline for five days and counted every day. Experiments were performed in triplicate. Full grey diamond  =  wild type at 30°C, full black diamond  =  wild type at 42°C, full grey square  =  temperature-sensitive clathrin mutant at 30°C, full black square  =  temperature-sensitive clathrin mutant at 42°C, full grey triangle  =  negative control (clathrin knockout cells) at 30°C, full black triangle  =  negative control (clathrin knockout cells) at 42°C.

Comparing growth rates of a genetically manipulated cell line with wild type usually provides valuable information on how severe the manipulation influences cellular functions globally. Here we used DKO-S cells transfected with wild type human clathrin heavy chain cDNA under a CMV promoter as a positive control, DKO-S cells with integrated E1584K-point mutated human clathrin heavy chain (cDNA) as well under a CMV promoter, and untransfected DKO-S cells as a negative control. To ensure that all genomic clathrin expression under the Tet-off promoter was prevented, we grew these cells in presence of doxycycline for four days prior to the actual assay, because existing clathrin gets degraded within this period (for an overview of the cell lines and conditions used see table 1) [17]. In the growth experiment for wild type cells we observed a slow growth rate at both permissive (30°C) and non-permissive (42°C) temperatures at nearly identical rates, whereas clathrin knockout cells first grew slowly and finally started to die at the permissive temperature (figure 2). At the non-permissive temperature the death of knockout cells was accelerated right from the beginning of the experiment which confirmed previous results [17]. In contrast, the TS-mutant cells behaved similarly to wild type at 30°C and similarly to the clathrin-depleted cells at 42°C although death was retarded by about one day (figure 2). This indicates that the TS-clathrin heavy chain is functional at the permissive but compromised at the non-permissive temperature.

Interestingly, when TS-mutant cells were left untouched for 24 h at the permissive temperature they began clustering. When these cells were shifted for further 24 h (or are lightly shaken) to the non-permissive temperature cells get separate to single cells or small clusters. A re-transfer for a further 24 h to the permissive temperature reversed this effect to some extend (supplementary figure S1). We hypothesise that the TS-clathrin is acting more slowly even under permissive-conditions, probably as a result of altered structural properties. Consequently, this could mean that membrane proteins like adhesive molecules get concentrated in slowly growing coated pits and have enough time to interact with a neighbouring cell. Therefore these cells stick together at the low temperature, whereas at the high temperature the phenotype of the TS-clathrin heavy chain is severely altered within 24 h and cell clustering is abrogated. This observation shows that the point mutation introduced into clathrin heavy chain, impacts the behaviour of DT40 cells in a temperature-dependent manner.

### Phenotypes of the mutated clathrin heavy chain

**Figure 3:**
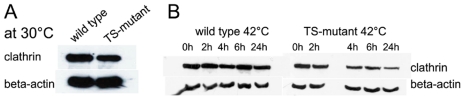
Phenotypes of mutated clathrin heavy chain. As previously described, cells were grown for four days at 30°C with doxycycline prior to the experiment. Aliquots of cells were incubated at 30°C or were shifted to 42°C. Samples of cell pellets were resuspended in lysis buffer and lysed at 4°C. A western blot was performed with equal amounts of protein. **A** Comparison of the amount of wild type clathrin heavy chain (wild type) and TS-clathrin heavy chain (TS-mutant) at 30°C (representative blot). **B** Clathrin heavy chain degradation at 42°C over 24 h for wild type clathrin heavy chain (wild type) and TS-clathrin heavy chain (TS-mutant). Samples were taken at 0, 2, 4, 6 and 24 h at 42°C (representative blot). Beta-actin served as a load control.

When TS-cells have been depleted of wild-type clathrin heavy chain expression under the Tet-off promoter, the amount of TS-clathrin heavy chain at 30°C under the CMV promoter is 72% of the wild type clathrin (figure 3A) and is not degraded within 24 h (data not shown). According to growth at the permissive temperature (figure 2), seemingly enough clathrin is available to carry out clathrin-dependent functions in the cell. In contrast, temperature-sensitive proteins are often misfolded at the non-permissive temperature and this generally leads to enhanced degradation if left at the non-permissive temperature for long periods of time [39], which was also seen with the TS-clathrin heavy chain. A representative western blot in figure 3B shows slow but continued degradation of TS-clathrin within 24 hours at 42°C, whereas the level of wild type clathrin was unaffected by prolonged exposure to 42°C. However, as experiments were carried out within the first 2 h after TS-clathrin inactivation at 42°C (unless otherwise noted) clathrin-loss within this time period is negligible.

As described above we have introduced the same point mutation in the human clathrin heavy chain as in one of the yeast TS-clathrin mutants, which failed to trimerize at the non-permissive conditions. Thus, we investigated whether the human TS-clathrin is also impaired in its ability to trimerize. We expressed C-terminal fragments of human clathrin heavy chain involved in trimerization (“hub” fragments) recombinantly in *E. coli* [40], [41]. We then purified the recombinantly expressed fragments and crosslinked them within the range of permissive to non-permissive temperatures (modified protocol of [42]). Unexpectedly, we did not observe a difference in the crosslinking pattern between wild type and TS-mutant (supplementary figure S2). In contrast to the corresponding yeast TS-clathrin it is possible that *in vitro* human TS-clathrin heavy chains can still trimerize.

### Clathrin localisation at the permissive and non-permissive temperature

**Figure 4:**
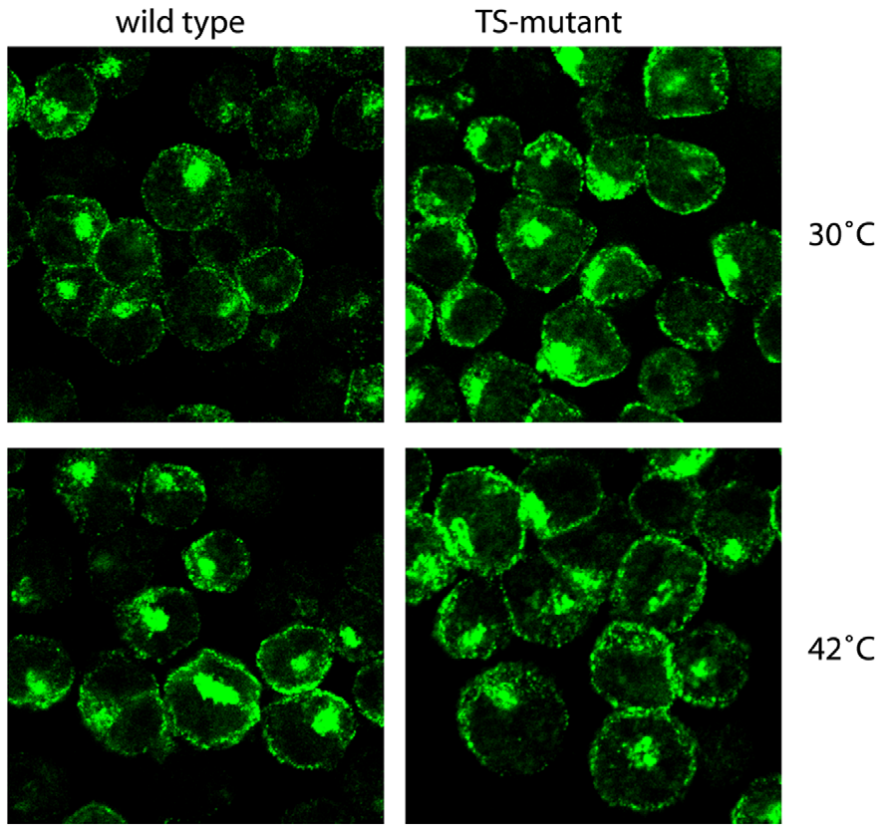
Clathrin localization at the permissive and non-permissive temperature. Cells were prepared as for the endocytosis assay. They were fixed with 4% paraformaldehyde, blocked for 1 h with blocking buffer (PBS with 0.3% TritonX-100, 0.05% saponin, 5% BSA) followed by an incubation with an anti-clathrin heavy chain antibody for 3 h and further with a FITC-conjugated secondary antibody (green) for 1–2 h. Images were taken and analysed using a confocal microscope. Cells show a typical clathrin stain visible as a dotted line at the plasma membrane and a prominent intracellular stain marking the Golgi apparatus.

At the non-permissive temperature, the yeast TS-clathrin with the point mutation E1590K fails to trimerise [36]. Having mutated the same residue as in this yeast clathrin heavy chain mutant we expected an altered clathrin staining pattern within the cells. To address this, wild-type and TS-mutant cells were incubated for a short period at the permissive or non-permissive temperature and subsequently stained for clathrin. Surprisingly, the typical clathrin pattern at the plasma membrane and on the Golgi apparatus was obvious in both wild type and TS-mutant, as well as at both temperatures (30°C and 42°C) (figure 4). Moreover there was not a significant difference between the fluorescent intensities of wild type and TS-mutant indicating that the TS-clathrin is still able to localize to its target membrane. Together with the crosslinking data this further indicates that our vertebrate TS-clathrin can still assemble at non-permissive conditions.

### Impaired transferrin endocytosis

**Figure 5:**
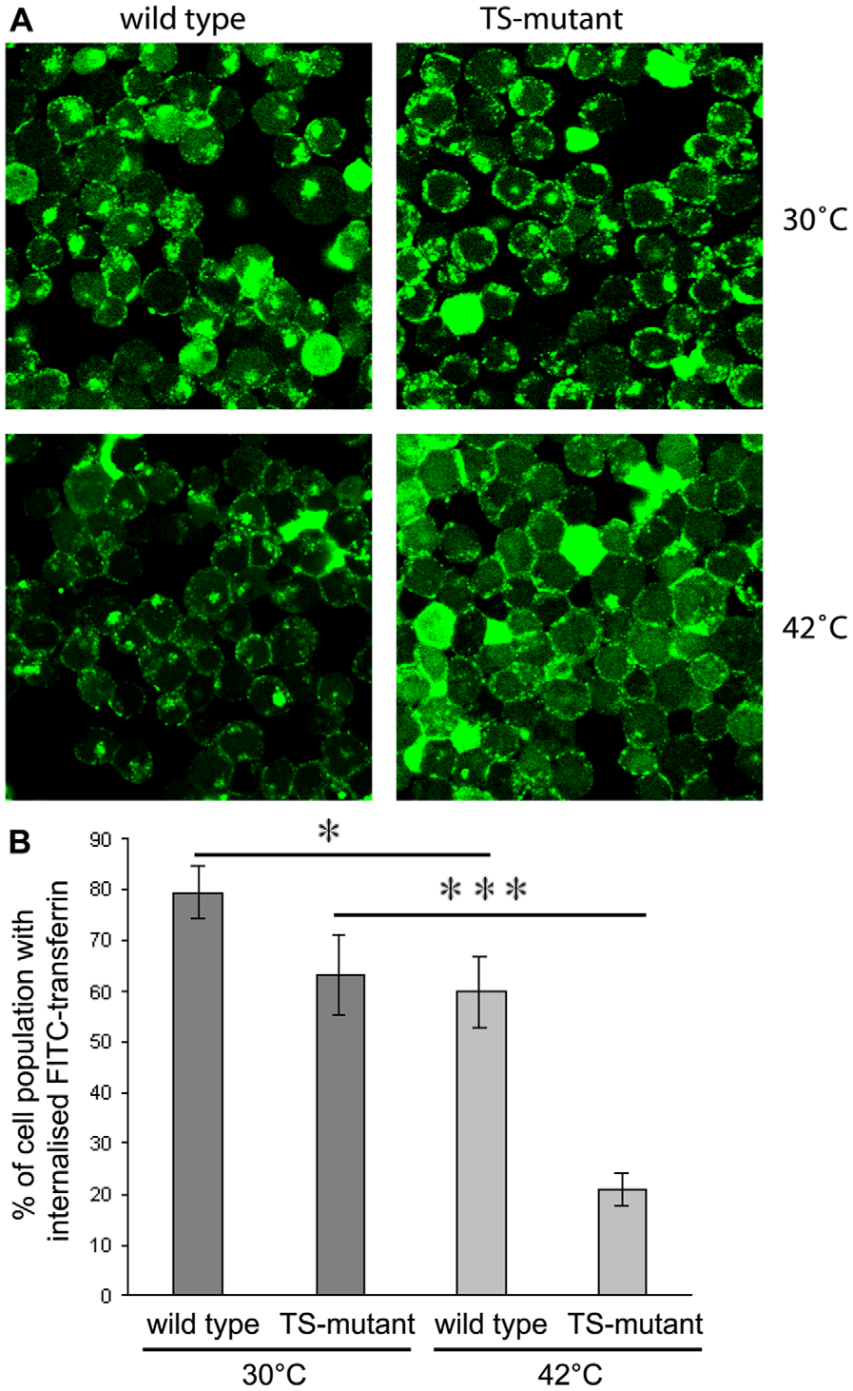
Endocytosis of FITC-transferrin at permissive and non-permissive temperature. Cells were grown at 30°C or shifted to 42°C for 30 min, washed with PBS and resuspended in serum-free medium. 30°C- and 42°C-preincubated cells were starved for iron for 30 min at 30°C or 42°C, respectively. For transferrin receptor loading, cells were pre-incubated with FITC- transferring at 4°C. For endocytosis cells previously incubated at 30°C were again incubated at 30°C, those incubated previously at 42°C shifted to 42°C for 90 min. Internalization was stopped by washing cells twice with ice-cold PBS. For analysis cells were spun on coverslips and fixed with 4% paraformaldehyde. Images were taken using a confocal microscope. All experiments were performed six times. **A** Cells with endocytosed FITC-transferrin visible as green prominent intracellular staining. **B** Calculating the percentage of the cell population capable of FITC-transferrin uptake (also see supplementary figure S2A–D) was done using the cell counter of ImageJ. Wild type cells at 30°C differ significantly (one star) from those at 42°C and TS-mutant cells differ highly significantly (three stars) from those at 42°C.

Down-regulation of clathrin-expression generally results in a severely reduced receptor-mediated endocytosis [5], [10], [11], [12], [13], [14], [17]. Classical markers to study clathrin-dependent endocytosis are uptake of transferrin and its receptor. We used FITC-labelled chicken transferrin (FITC-transferrin) to follow its uptake into the cell by immuno fluorescence (figure 5A). At the permissive temperature (30°C), all cells treated with FITC-transferrin stained the cell surface. In TS-cells this stain seems to be more prominent than in the wild type. Furthermore, at the permissive temperature 80% of wild type cells and 63% of the TS-mutant cells show a distinctive perinuclear internal staining presumably representing multiple endosomal compartments containing FITC-transferrin [43] as has been observed before [44]. At the non-permissive temperature, this uptake was significantly reduced: whilst most cells continued to bind transferrin at the surface, 60% of the wild type cells but only 21% of the TS-mutant cells internalised transferrin (figure 5B and supplementary figure S3A–D). The reduced uptake rate of FITC-transferrin for wild type cells at the non-permissive temperature was confirmed by a FACS-based endocytosis assay (supplementary figure S4). However for the TS-mutant no altered uptake rate compared to wild type at 42°C was observed by FACS which conflicts with the immuno fluorescence data. This is most likely due to the inability of the FACS analysis to discriminate between FITC-transferrin bound to receptors on the plasma membrane and intracellular transferrin within endocytotic vesicles (figure 5A). For the FACS assay, wild type and TS-mutant cells had the same amount of FITC-transferrin at their surface. However, wild type cells began to endocytose it whereas in the TS-mutant the FITC-transferrin remained stuck on the plasma membrane as judged by immuno fluorescent images (figure 5A). For the duration of the assay both cell types contained similar total amounts of FITC-transferrin, although it was located differently, and consequently not distinguished by the FACS machine. Yet, why wild type cells have a decreased endocytosis rate at the non-permissive temperature is not clear. One could speculate that *human* wild type clathrin heavy chain is slightly impaired at the non-permissive temperature and this effect is enhanced in the human TS-clathrin mutant carrying the E1584K point mutation. In contrast to humans, birds usually run their body temperature higher than 37°C [45], [46]. All other (chicken) proteins of the CCV-machinery are unlikely to be affected at the non-permissive temperature.

### Localisation of FITC-transferrin and transferrin receptor at permissive and non-permissive temperature

**Figure 6:**
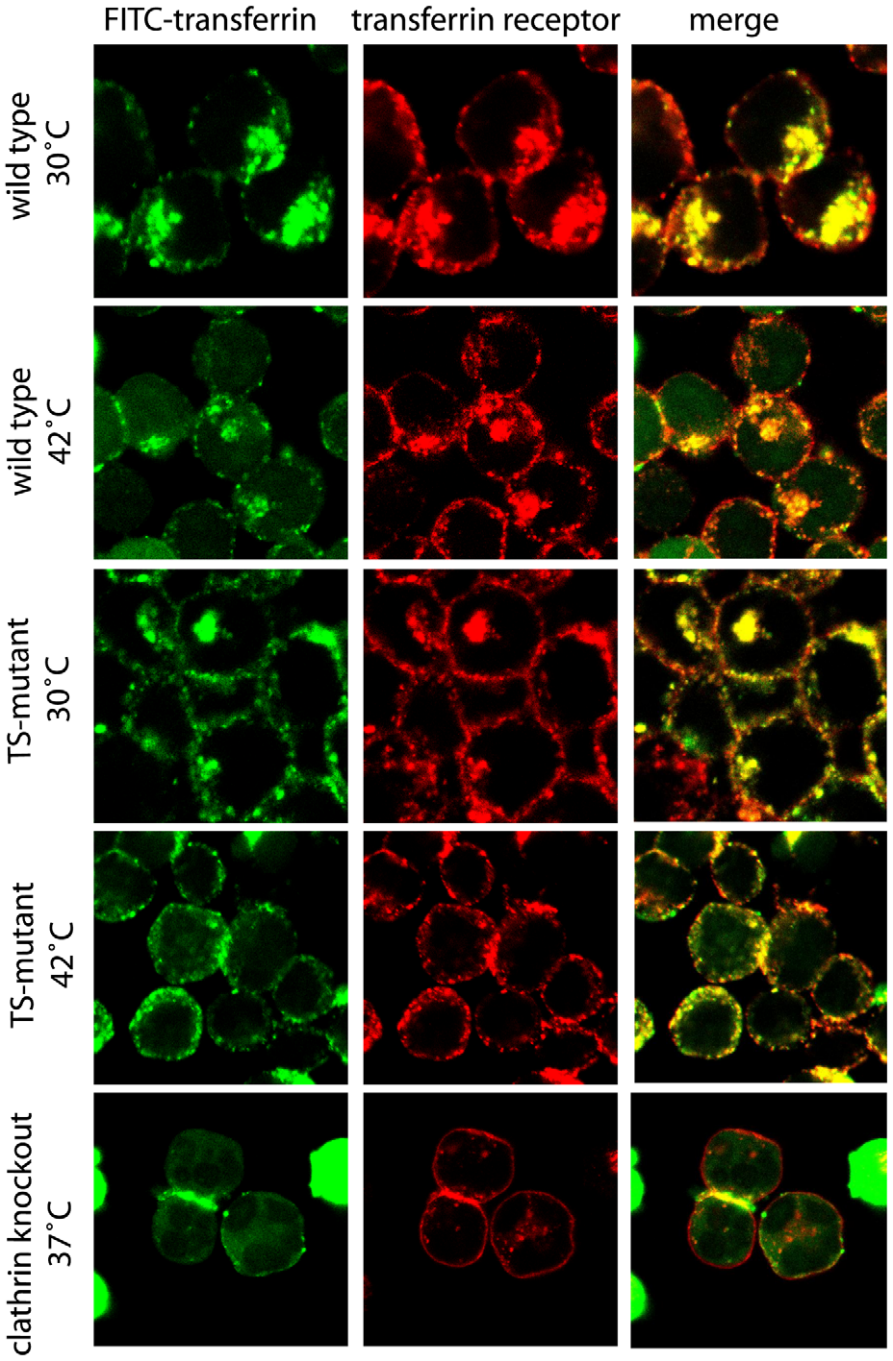
Endocytosis of FITC-transferrin and transferrin receptor at permissive and non-permissive temperature. The endocytosis assay with FITC-transferrin (green) was carried out as described before in figure legend 5 however followed by an indirect immune stain using an anti-transferrin receptor antibody as a primary antibody and a Cy3-conjugated secondary antibody (red). A confocal microscope was used to take images and to analyse them. For clathrin *knockout* cells the assay was performed at 37°C because this strain died under the conditions (30°C and 42°C) used for wild type and TS-mutant.

The impaired uptake of transferrin in TS-mutant cells at the non-permissive temperature could be caused by a mislocalised transferrin receptor. Using the endocytosis assay as described before, transferrin receptor in wild type cells was detected at the plasma membrane as well as internally as a prominent intracellular stain at both permissive and non-permissive temperatures. This prominent internal stain, probably representing accumulated endosomes [43], shows major co-localization with FITC-transferrin (figure 6). However, co-localization of transferrin receptor with transferrin was not very intense on the plasma membrane, because only a small amount of FITC-transferrin was localised here. The TS-mutant at the permissive temperature showed co-localization of FITC-transferrin and its receptor at both the plasma membrane and internally, but at 42°C, this distribution significantly shifted such that there was only a strong co-localization at the plasma membrane. This is fully consistent with the inhibition of receptor-mediated endocytosis in TS-mutant cells at the non-permissive temperature (figure 5). As the transferrin receptor is located correctly at the plasma membrane in TS-mutant cells at 42°C, impaired endocytosis is a direct consequence of the temperature-sensitive effect of the mutant clathrin. Interestingly in clathrin knockout cells, transferrin receptor is visible as a sharp (red) line at the plasma membrane and as few small spots inside the cell. Yet, hardly any FITC-transferrin is visible (figure 6) and thus it seems that clathrin knockout cells cannot bind transferrin efficiently without clathrin.

### Regular sorting of the lysosomal enzyme beta-glucuronidase

**Figure 7:**
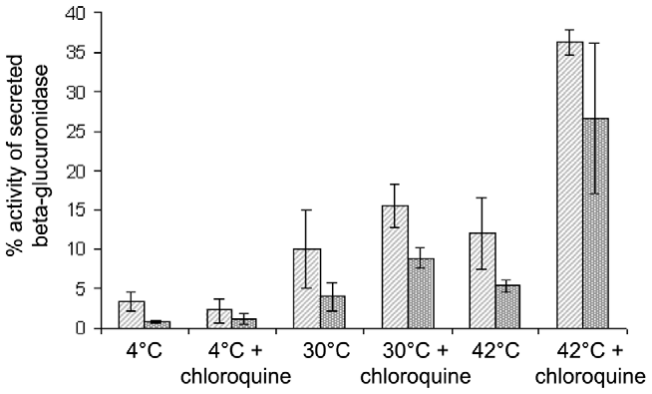
Impaired trafficking from the late Golgi apparatus to the lysosomes: Secretion of beta-glucuronidase as an example of lysosomal enzymes. Aliquots of cells were incubated for 2 h at either 30°C or 42°C as indicated. To some aliquots chloroquine was added. After incubation cells were harvested and supernatant and cell pellet were separated. The cell pellet was lysed in an equal volume as the supernatant. 50 µl of both supernatant and lysate were transferred to 200 µl substrate buffer in a black 96 flat bottomed 96 well plate. Catalysis of the beta-glucuronidase was determined using a fluorescent plate reader (excitation filter  = 380−12 nm, emission filter  = 460 nm). For kinetics, values were measured every minute over 100 min at 37°C. The calculated straight provided the basis for determination of the slope/min using the software of the manufacturer to determine the percentage activity of secreted beta-glucuronidase. 100% beta-galactosidase activity was calculated from the activity of the supernatant (secreted beta-glucuronidase) and cell pellet (intracellular beta-glucuronidase). Experiments were performed in triplicates. The graph shows a comparison of wild-type and TS-mutant at permissive and non-permissive temperature. Striped, light gray bars  =  wild type, punctated, dark gray bars  =  TS-mutant.

Clathrin at the trans-Golgi network (TGN) has been implicated in sorting of lysosomal proteins *via* the mannose-6-phosphate receptor [5]. Blocking this pathway results in the rapid secretion of newly synthesised lysosomal enzymes rather than trafficking to the lysosome [47]. Previously, it was shown that clathrin-depleted DKO-R cells did not secrete lysosmal enzymes [17] indicating that TGN-lysosome traffic is unaffected. To test whether this absence of mis-secretion reflected an adaptation to clathrin-depletion, we measured the beta-glucuronidase activity [48] secreted by TS-cells after only 2 h of incubation at the permissive or non-permissive temperature. For comparison, cells were incubated in the presence of the lysosomal acidification inhibitor chloroquine, forcing TGN derived vesicles to secrete their contents into the medium [47], [49], to estimate the upper limit for the potential effect. Although endocytosis is severely reduced at the high temperature, we supplemented the assay with mannose-6-phosphate to block re-uptake of secreted beta-glucuronidase. The graph in figure 7 shows a general tendency for enhanced beta-glucuronidase secretion with raising temperature and when adding chloroquine to the reaction. Beta-glucuronidase activity is given in% of secreted beta-glucuronidase activity (with 100% activity consisting of secreted and intracellular beta-glucuronidase). There was about 10% secretion for wild type and TS-clathrin mutant at 30°C and about 15% β-glucuronidase activity in the supernatant with chloroquine-treatment at the same temperature (figure 7). At the non-permissive temperature the values for wild type and TS-mutant show slightly higher beta-glucuronidase activity but still no significant difference between these cells could be observed. Hence, there is no evidence for missorting of beta-glucuronidase in TS-mutant cells at 42°C although secretion percentages of 35% for the wild type and 25% for the TS-mutant at the non-permissive temperature would be possible as judged by cells treated with chloroquine (figure 7).

### Lack of detectable compensation through alternative endocytic pathways

**Figure 8:**
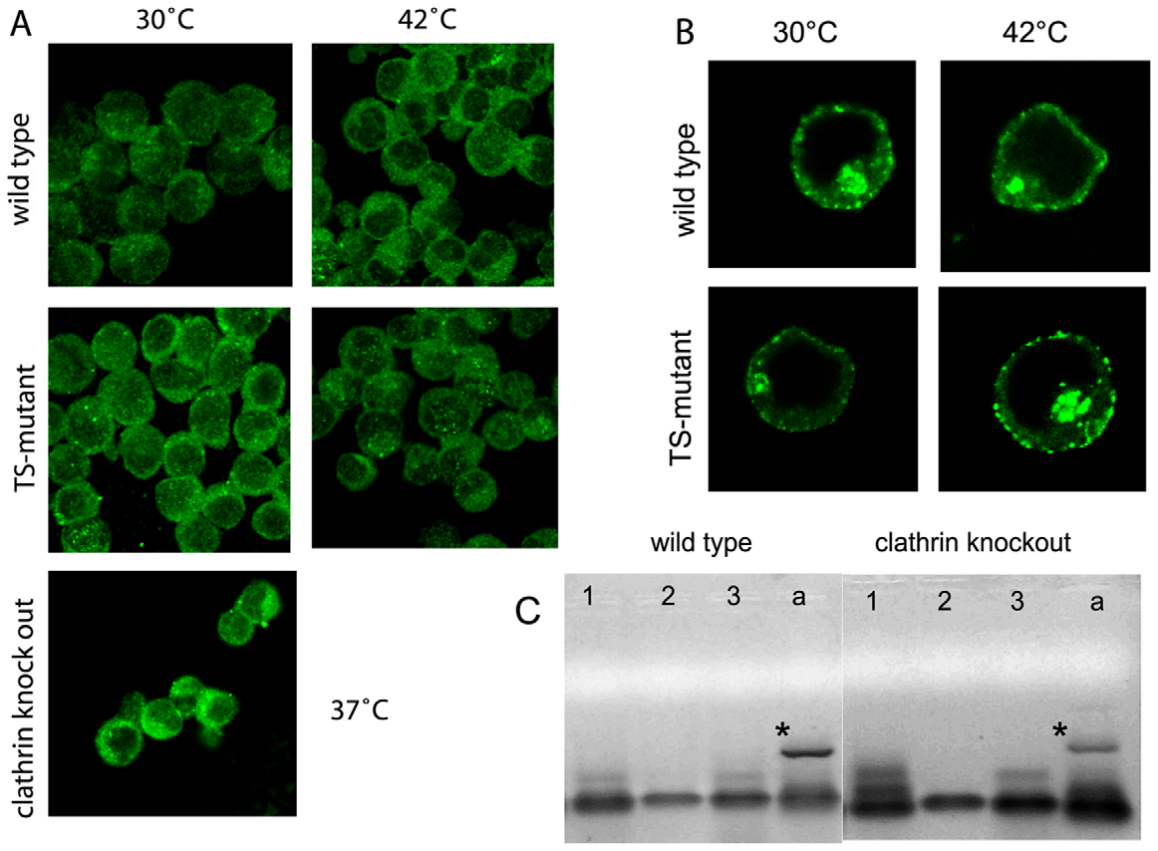
Effects of clathrin inactivation on cellular function. **A** Actin localisation in wild type, TS-mutant and clathrin *knockout* cells at permissive and non-permissive temperature. The assay was carried out as described in figure legend 4, but stained for actin (green) instead. **B** GFP-Eps15 (green) distribution in wild type and TS-mutant at permissive and non-permissive temperature. The assay was performed as explained in figure legend 5 (endocytosis assay), however, no immune stain was performed. **C** RT-PCR for caveolins. Total RNA was extracted from wild type and clathrin *knockout* cells and a RT-PCR followed by a PCR for all three caveolin homologues (caveolin-1  = 1, caveolin-2  = 2, caveolin-3  = 3) and beta-actin as positive control (a; marked with an asterix) has been performed.

Clathrin-dependent endocytosis is the major constitutive endocytic route in eukaryotic cells although other key proteins can mediate endocytosis [50]. For example actin is involved in both clathrin-dependent and -independent endocytic pathways [30] [51]. Specially in the cell line DKO-S it has been shown that in clathrin-depleted cells actin is partly responsible for B-cell receptor uptake [52]. For that reason we expected that inactivating the clathrin-dependent pathway would have downstream effects on the actin response. Surprisingly, no difference of the actin distribution for either short-term (TS-mutant at 42°C) or long-term (clathrin knockout cells) clathrin depleted conditions could be observed (figure 8A). This suggests that actin-based pathways do not compensate if the clathrin-dependent endocytosis pathway is impaired.

Caveolin is the major component of another very well described endocytic pathway, the so-called caveolae-dependent endocytic pathway, the existence of which in B-cells is controversially [53], [54], [55], [56], [57]. Three caveolin isoforms are known in humans (and have homologues in chicken) and are expressed in different types of tissue. To assess the possibility that B-cells up-regulate one of the caveolins to compensate for chronic clathrin loss, RT-PCR for all caveolin homologues in chicken were performed but showed no mRNA up-regulation in clathrin knockout cells (figure 8B). This result also indicates that in chicken B-lymphocytes caveolae are absent, making it impossible for them to compensate for clathrin-depletion.

Another possibility for a non-functional clathrin-dependent pathway may be an incomplete CCV- machinery. To address this issue we focused on Eps15 as a representative example of an accessory protein to focus on. Eps15 is usually co-localized with AP-2 at the plasma membrane or AP-1 at the TGN [58] but is excluded from CCVs, but its role in CCV formation has not yet been defined [59], [60]. We found that the overall distribution of the GFP-Eps15 in transfected cells is the same for wild type and TS-mutant at both permissive and non-permissive temperatures (figure 8C) at the plasma membrane and TGN. However, it seems that Eps15 is more abundant at the plasma membrane of the TS-mutant. Nevertheless, Eps15 is correctly localized at the plasma membrane and the Golgi apparatus, which is consistent with the usual clathrin localisation shown in figure 4 for the permissive and non-permissive temperature. We can conclude that the effects of clathrin-dependent endocytosis are a direct consequence of the TS-effect of the mutant clathrin.

Our results show that clathrin-dependent processes stop when clathrin is rapidly inactivated and do not continue by missorting or mislocalization of vesicles or proteins, respectively.

## Discussion

Investigating clathrin-dependent processes in vertebrates relies on clathrin knockdown techniques, because no inhibitor is known for clathrin. Complete clathrin degradation can take several days [17], [21] and hence, within this time an up-regulation of compensatory endocytic pathways may occur. To separate short-term and long-term consequences of clathrin inactivation in higher eukaryotes, we have successfully engineered the first vertebrate clathrin heavy chain temperature-sensitive mutant. This is supported firstly by the decreased growth rates of the TS-clathrin mutant over several days at the non-permissive temperature underlining that clathrin function is severely restrained under these conditions. Secondly, the mutated clathrin heavy chain is degraded within several hours indicating a structural defect of the protein at the high temperature. Thirdly, and most importantly, the uptake of transferrin was severely impaired at the non-permissive temperature revealing an immediate effect of clathrin inhibition when shifted to the high temperature.

Despite these obvious phenotypes of the TS-clathrin mutant under non-permissive conditions, we conclude that the E1584K mutation does not cause dissociation of clathrin-triskelions when shifted to the high temperature, since clathrin and Eps15 showed no altered localization compared to wild type under these conditions. Supportingly, our crosslinking data showed no difference between wild type and TS-clathrin under these conditions either. This behavior is in contrast to the corresponding yeast TS-clathrin which failed to trimerize at the permissive and non-permissive temperature [36]. In contrast, another yeast TS-mutant with a truncated C-terminus [23] forms stable triskelions at the non-permissive temperature, although it was temperature-sensitive for growth. As these authors, we favor the idea that the vertebrate TS-clathrin phenotype is based on an altered lattice- and vesicle-formation [23] and triskelions which have already formed at the permissive temperature remain stable when shifted to the non-permissive temperature.

However, the TS-mutant is not simply an immediate clathrin knockout, since there is a significant difference between these two cell types: Immuno fluorescent data illustrate a co-localisation of transferrin and transferrin receptor for TS-mutant cells under non-permissive conditions, whereas in clathrin knockout cells no transferrin could be bound by the transferrin receptor at the plasma membrane. This suggests an altered composition or organization of the plasma membrane proteins in clathrin knockout cells. Most likely, pit- and vesicle-formation in TS-cells is less efficient, slower and possibly slightly different to wild type because the mutated clathrin heavy chains may assemble in structurally altered triskelions.

With our novel tool in hand to investigate clathrin-dependent functions within a cell and without changing the protein composition at the target membranes we studied intracellular trafficking at the non-permissive temperature. Surprisingly, we did not observe a missorting of lysosomal enzymes, nor alterations of the actin cytoskeleton under both long- and short-term clathrin inactivation conditions, nor saw we compensation by other endocytic mechanisms such as up-regulation of caveolins. In contrast to our vertebrate system, where clathrin-dependent processes did not continue after clathrin-inactivation, the corresponding yeast TS-clathrin mutant showed immediate effects such as missecretion of alpha-factor (mating pheromone) and missorting of the lysosomal protein carboxypeptidase Y [26], [36], [38]. This emphasizes not only a difference in the requirement for clathrin in yeast, but also a different cellular response.

The chicken B-lymphocyte cell line DT40 relies on clathrin to internalize transferrin. Besides this, other processes such as TGN-lysosome traffic do not similarly rely on clathrin as they are functional under conditions when clathrin-mediated endocytosis is blocked. This is consistent with DT40 clathrin knockout cells, where also no missorting of the lysosomal proteins could be detected [17]. Our assays could not identify any compensatory for the actin- or other clathrin-independent endocytic routes when clathrin was inhibited by the non-permissive conditions.

Here we present the first rationally designed vertebrate temperature-sensitive clathrin heavy chain mutant showing an immediate phenotype at the non-permissive temperature. This vertebrate TS-allele of clathrin may serve in future as a valuable tool to investigate in detail clathrin-dependent and independent trafficking events in vertebrate such as lysosomal trafficking or the role of actin in endocytosis.

## Materials and Methods

### Creation of cell lines

We created stable cell lines expressing either wild type human clathrin cDNA or human temperature-sensitive clathrin (TS-clathrin), by using human clathrin heavy chain cDNA under a constitutive CMV (cytomegalovirus) promoter (verified full length cDNA clone IRATp970E0575D from RZPD  =  Deutsches Resourcenzentrum fuer Genomforschung GmbH; accession number: Uniprot Q00610). A single point mutation was set *via* site-directed mutagenesis using the primers 5′-CCTAAAAACTGCATGGAGGCACAATATC-3′ and 5′- GCAGTTTTTAGGACGACATCTGGCCTTAAAAG-3′ leading to the amino acid E(1584)–K substitution on protein level (sequence confirmed by sequencing). The plasmids pCMVSport6humanCHC (expressing wild type clathrin heavy chain) and pCMVSport6humanTSCHC (expressing temperature-sensitive clathrin heavy chain) were transfected *via* electroporation into DKO-S cells [17]. The cell lines were grown in presence of 1 µg/ml doxycycline in RPMI1640 supplemented with 10% bovine calf serum and with 0.3% chicken serum at 37°C for one day and were then transferred to 30°C for two days in a 5% CO_2_ atmosphere. Cells were diluted to one cell per well in a 96 well plate. Wettey *et al*. showed that under low chicken serum conditions DKO-S cells die without clathrin. Thus, wild type and TS-clathrin heavy chain cDNA transfected cells were selected under low-serum and low-temperature conditions for growth, because only those expressing functional clathrin could survive. Single clones were tested for growth deficiency at 42°C and for clathrin expression *via* indirect immune fluorescence stain for clathrin heavy chain. For experiments we used the cell lines listed in table 1. Except where indicated otherwise, in all further experiments cells were grown in RPMI1640 medium supplemented with 10% bovine calf serum, 1% chicken serum and 1 µg/ml doxycycline in a 5% CO_2_ atmosphere. *Note*: To avoid clustering of temperature-sensitive cells light shaking every day is recommended. We also observed that prolonged propagation of the cells leads to a loss of the TS-effect.

### Growth at permissive and non-permissive temperature

To prepare cells for the growth experiment, the cell lines DKO-S wild type (cells with wild type clathrin heavy chain expressing under a Tet-off promoter [17] plus randomly inserted clathrin heavy chain under a CMV-promoter), original DKO-S cells (with wild type clathrin heavy chain expressing under a Tet-off promoter) and TS-mutant cells (wild type clathrin heavy clathrin expression under a Tet-off promoter and TS-clathrin heavy chain under a CMV promoter) were grown for four days at 30°C with doxycycline to repress clathrin heavy chain expression under the Tet-off promoter (table 1). Within this period the remaining clathrin was allowed to be degraded [17]. To start the experiment, viable cells were counted using trypan blue stain and were diluted to 1×10^5^ cells/ml into fresh medium. A volume of 10 ml for each cell line was incubated at 30°C (permissive temperature) or 42°C (non-permissive temperature) in presence of doxycycline for five days and counted every day. Permissive and non-permissive temperatures were determined previously within a temperature range of 30°C to 45°C.

### Clathrin degradation

Wild type and TS-clathrin expressing cell lines were grown for four days with doxycycline at 30°C. One aliquot of the cells were kept on 30°C the other half was shifted to 42°C. Samples of 50 ml were taken at 0, 2, 4, 6 and 24 h spun immediately (1100 rpm, 8 min) and washed with PBS (phosphate-buffered saline). Pellets were resuspended in 100 µl lysis buffer (PBS, 0.1% Triton X-100, protease inhibitors (complete No. 11697498001, Roche) and lysed by vigorously shaking at 4°C for 30 min followed by a 30 min incubation with 1 µl DNAse (Promega, 1 U/µl) at RT (room temperature). Samples were spun at top speed at 4°C for 30 min and the supernatant was retained. Protein content was determined by a BCA (bicinchoninic acid) assay (Thermo Scientific). Equal amounts of protein were loaded on an 8% Laemmli gel and blotted on a PVDF (polyvinylidene fluoride) membrane.

### Western blot

Proteins were loaded on an 8% Laemmli gel and blotted on a PVDF membrane. Blots were blocked with 5% semi-skimmed milk powder in PBS for 30 min at RT and cut in appropriate pieces as judged by the molecular weight standard. Slices with high molecular weight proteins were incubated with an anti-clathrin antibody (Abcam ab21679, 1∶200) and slices with the low molecular weight proteins with an anti-actin antibody (A066, Sigma, 1∶1000) both in 5% semi-skimmed milk powder in PBS at 4°C overnight. A HRP-coupled (horseradish peroxidase) secondary antibody (A0545, Sigma, 1∶8000) in 1% semi-skimmed milk powder in PBS was used for 2 h incubation and protein fragments were visualised with ECL-reagents (enhanced chemiluminescent; Thermo Scientific).

### Crosslinking of truncated triskelions

Recombinant expression of “clathrin-hub fragments” (C-terminus of clathrin involved in trimerization) was based on the protocol described in Ybe et al. and Liu et al. [40], [41]. Hub-fragments were created via PCR with the primers 5′-GCCATTGGATCCAAATTTGATGTCAATACTTC-3′ and 5′- GGCTTTGGGTACAGCATGTGAAAGCTTGCGATA-3′ and wild type or TS-clathrin human cDNA serving as a template. Briefly, *E. coli* BL21 DE3 was freshly transformed with pET28a human clathrin wild type hub or pET28a human clathrin TS-hub fragment. Over-night cultures were diluted 1∶30 in fresh LB medium and grown for 2 h at 37°C. Protein expression was carried out at 30°C with IPTG (100 mg/ml) for 3 h. Bacteria were sonicated in ice cold buffer A (20 mM Na2HPO4, 10 mM imidazole, 0.5 M NaCl, pH 7.4), and subsequently supplemented with lysozyme, protease inhibitors (Roche) and 20 mM beta-mercaptoethanol (final concentration). The lysate was loaded onto a nickel affinity column (Sepharose Fast Flow) charged with NiSO_4_ and washed extensively. For elution a 1∶1 mixture of buffer A and buffer B (same as A but with 0.5 M imidazole) was used. Samples were further purified by size exclusion chromatography on a Superdex 75 column equilibrated in 10 mM Tris pH 7.9 buffer. The protein content was determined by the BCA assay. Crosslinking was carried out using 1 µM clathrin hub fragment (wild type or TS-mutant) in PBS plus 10 mg/l BS3 crosslinker (bis[sulfosuccinimidyl]suberate; Thermo scientific) for 5 min at indicated temperatures. The reaction was terminated by addition of 4× sample buffer (NuPAGE, Invitrogen) supplemented with 40 mM Tris/Cl pH 7.4. 20 µl of each sample was run on a 4–12% SDS PAGE gel (NuPAGE, Invitrogen) and stained with silver or a Western blot was performed using an anti-His-tag antibody as a secondary and ECL reagents was used for visualisation (modified protocol of [42]).

### Indirect immune fluorescence

An appropriate amount of cells was harvested, washed with PBS, spun (350 rpm, 3 min, RT) on 0.05% polylysine-coated coverslips and fixed with 4% paraformaldehyde for 30 min. Cells were further washed with wash buffer (PBS with 0.3% TritonX-100 and 0.05% saponine) and blocked for 1 h in blocking buffer (wash buffer with 5% BSA). Then cells were incubated with primary antibody in a blocking buffer solution for 3 h at RT. Next, cells were intensively washed and incubated with the secondary antibody in blocking buffer for 1–2 h at RT. After washing, nuclei were stained with Hoechst 33342 or Propidium iodide, dried and covered with mounting medium (Vectashield, H-1400). The following antibody concentrations were used: Anti-clathrin heavy chain (Abcam ab21679) 1 µg/ml, anti-actin (Sigma A066) 1∶100, anti-transferrin receptor (1 mg/ml; mouse anti-chicken transferrin receptor antibody was a generous gift from Prof. Anne Mason, Department of Biochemistry, The University of Vermont, USA) 1∶250, anti-rabbit-FITC (Sigma F7512) 1∶150, anti-rabbit-IgG-TRITC (Sigma T2527) 1∶70, anti-mouse-Cy3 (Jackson Immuno Research Laboratries, 115-165-003) 1∶80, anti-mouse-FITC (Sigma F0257). Evaluation of the preparations was done using a fluorescent microscope (Axioskop, Zeiss) and a confocal microscope (Olympus Fluoview IX81).

### Fe-loading and FITC-labelling of chicken transferrin

For iron (Fe) loading 10 mg conalbumin (chicken transferrin; Sigma) was incubated in 1 ml of a 10 mg/ml Ferricammonium citrate - bicarbonate (0.05 M) solution pH 9.6 for 1 h. For FITC-labelling (fluorescein isothiocyanate), 1 ml of this solution was added to 1 ml of 1.5 mg/ml FITC in PBS pH 7.2 solution and incubated for 30 min at RT. The labelling reaction was stopped by adding 1 ml of 500 mM NH_4_Cl and incubated for 45 min at RT. Fe-loaded conalbumin was 1-fold and Fe-FITC-labelled conalbumin was purified 2-times using a PD10 column. The concentration of the Fe-loaded conalbumin was approx. 5 mg/ml and of the FITC-labelled conalbumin approx. 0.5 mg/ml.

### Endocytosis assay

To inactivate TS-clathrin, 2×75 ml of 2.5×10^5^ cells/ml were aliquoted and either kept at 30°C or shocked at 42°C for 30 min in a water bath. Cells were harvested (1100 rpm, 8 min), washed with PBS and resuspended in 1 ml RPMI1640. 30°C- and 42°C-preincubated cells were starved for iron in a 5% CO_2_ atmosphere for 30 min at 30°C or 42°C, respectively. After harvesting pellets were resuspended in 0.5 ml ice-cold PBS and kept on ice for 10 min. Next either 150 µl Fe-FITC-transferrin or 100 µl Fe-transferrin was added and incubated for further 5 min on ice. For uptake cells previously incubated at 30°C were again incubated at 30°C, those incubated previously at 42°C shifted to 42°C for 90 min in a 5% CO_2_ atmosphere. One aliquot was kept on ice. Then cells were harvested, washed twice with ice-cold PBS and resuspended in 350 µl PBS. Cells were spun (350 rpm, 3 min, RT) on 0.05% polylysine-coated coverslips and fixed with 4% paraformaldehyde. Cells were either washed with PBS and nuclei were stained with Hoechst 33342 (bis-Benzinide), dried and covered with mounting medium (Vectashield H-1400) or an indirect immunofluorescent stain was performed. Where necessary, calculating of endocytosed FITC-transferrin was done using the cell counter of ImageJ (http://rsb.info.nih.gov/ij/).

### Quantitation of FITC-transferrin uptake

Cells (grown for four days at the permissive temperature, TS-mutant with doxycycline) were harvested by centrifugation (1200 rpm, 7 min), washed and starved of iron by incubation in a 5% CO_2_ atmosphere at 30°C for 30 min in serum-free RPMI 1640. After harvesting, cells were resuspended in PBS at a concentration of 2×10^7^ cells/ml and added to wells of a 96 well plate in 75 µl aliquots. Plates were pre-incubated at either 30°C or 42°C in a 5% CO_2_ atmosphere for 20 min, then 25 µl Fe-FITC-transferrin (150 µg/ml) was added to each well and plates returned to 30°C or 42°C. Aliquots were harvested at 0, 5, 15 and 30 min time-points after Fe-FITC-transferrin addition and added to 0.5 ml ice cold PBS containing 50 µM deferoxamine mesylate (Sigma) to scavenge uninternalized transferrin. Fe-FITC-transferrin uptake was analysed using a FACScan flow cytometer (Becton Dickinson). For each sample 10,000 cells were analysed in triplicate. Quantitation was carried out using Summit 4.3 software (Beckman Coulter).

### Secretion assay

An appropriate amount of cells grown at 30°C was harvested (1100 rpm, 8 min) and washed twice with PBS. The pellet was resuspended in RPMI1640 without phenol red but supplemented with 1% BSA and adjusted to 5×10^6^ cells/ml. Aliquots of 500 µl were incubated for 2 h at either 30°C or 42°C in a 5% CO_2_ atmosphere. All aliquots were supplemented with mannose-6-phosphate (10 mM final concentration). To some aliquots chloroquine was added (final concentration 500 µM). After incubation, cells were harvested and supernatant and cell pellet were separated. The cell pellet was lysed in an equal volume as the supernatant with lysis buffer (PBS, 0.1% Triton-X100, protease inhibitors (complete No. 11697498001, Roche)) by vortexing for 5 sec. 50 µl of both supernatant and lysate were transferred to 200 µl substrate buffer (1 mM 4-Methylumbelliferyl-β-D-Glucuronide in 100 mM sodium citrate pH 5.5 with 0.1% Triton-X100) in a black 96 flat bottomed 96 well plate. Catalysis of the beta-glucuronidase was determined using a fluorescent plate reader (Fluostar optima, BMG Labtech: Excitation filter  = 380−12 nm, emission filter  = 460 nm) (modified protocol of [61]). For kinetics samples were automatically measured every minute over 100 min at 37°C (gain adjustment 50%). From these results a straight was analyzed of which the slope/min was calculated using the software of the manufacturer to determine the percentage activity of secreted beta-glucuronidase. 100% beta-glucuronidase was calculated from the activity of the supernatant (secreted beta-glucuronidase) and cell pellet (intracellular beta-glucuronidase).

### RT-PCR

Total RNA from cells was isolated using the protocol for cells of the “Absolute RNA RT-PCR Miniprep-kit” (Stratagene). The reverse transcription of mRNA into cDNA was performed using SuperScriptII RNaseH reverse transcriptase (Invitrogen) and random primers (Promega). Fragments of choice were amplified from the obtained cDNA using Taq-polymerase and were analysed on an agarose gel. The following primers were used: For caveolin-1 5′-TGGAAGGCCAGTTTTACCAC-3′ and 5′-CCAGGTTGATGGTGCTTTTT-3′, for caveolin-2 5′-TTCGTTGCACCATTCTTTCA-3′ and 5′-CAGTGAGGGCCAAAAATGAT-3′, for caveolin-3 5′- GCAGAGAGAGCTGGAGGAGA-3′ and 5′- CCAGTACTTGCTGACGGTGA-3′, beta-actin 5′-ATTCCTATGTGGGCGACGAG-3′ and 5′-TGGATAGCAACGTACATGGC-3′.

### Transfection of a GFP-Eps15 construct

The GFP-Eps15 (pEGFP-EΔP/C) [59] construct was transfected by electroporation in either wild type or TS-clathrin expressing cells both grown at 30°C with doxycycline for four days. Transfected cells were incubated at 30°C with doxycycline for approx. 48 h and the endocytosis assay with Fe-conalbumin described above was carried out at 30°C and 42°C. Then cells were spun (350 rpm, 3 min, RT) on 0.05% polylysine-coated coverslips, fixed with 4% paraformaldehyde for 30 min, washed with PBS, stained with Hoechst 33342, dried and mounted with mounting medium.

### Monitoring cell clustering

DKO-S wild type and TS-mutant cells (treated with doxycycline) were grown for four days at the permissive temperature. Of these cells 1 ml (2.5×10^4.^cells) was transferred to a 24 well-plate and incubated for 24 h at 30°C in a 5% CO_2_ atmosphere. Then cells were shifted to 42°C for another 24 h followed by a re-shift for 24 h to the permissive temperature. Cell aggregation was followed using a microscope and representative images were taken.

## Supporting Information

Figure S1 In vitro crosslinking of truncated clathrin triskelions. Purified clathrin heavy chain “hub”-fragments (C-terminus of clathrin heavy chain involved in trimerization) were incubated within a temperature range of 26–47°C for 5 min supplemented with crosslinker. The crosslinking reaction was stopped by adding 4× SDS-PAGE-sample buffer. An equal amount of protein was loaded on a SDS-gradient gel followed by a silver stain. 1 = 25.9°C, 2 = 26.1°C, 3 = 27.2°C, 4 = 28.9°C, 5 = 31.2°C, 6 = 33.8°C, 7 = 36.6°C, 8 = 39.4°C, 9 = 42.0°C, 10 = 44.2°C, 11 = 45.9°C, 12 = 46.8°C, not = no crosslinker added.
(3.19 MB TIF)

Figure S2 Images to calculate endocytic cells. The endocytosis assay was performed as decribed in figure legend 5. The six individual confocal images 2A–D were used to calculate (cell counter of software ImageJ) the percentage of the cell population which had a prominent intracellular staining, the endocytosed FITC-transferrin. A summary of the results is shown in figure 5B. A = wild type 30°C, B = wild type 42°C, C = TS-mutant 30°C, D = TS-mutant 42°C.
(6.82 MB TIF)

Figure S3 FACS-based quantification of FITC-transferrin endocytosis. Cells were grown for several days at 30°C (TS-cells with doxycycline). For the assay, cells were starved for iron followed by a pre-incubation with FITC-transferrin. Then cells were aliquoted and shifted to either 30°C or 42°C. Over indicated time points cells were analysed via FACScan flow cytometer. Values were normalized against starting value. Blue diamond = wild type 30°C, red triangle = wild type 42°C, green square =  TS-mutant 30°C, orange cross = TS-mutant 42°C.
(5.78 MB TIF)

Figure S4 Clustering of TS-mutant cells at the permissive temperature after 24 h of incubation. Clustering of cells grown untouched under permissive conditions for 24 h, followed by shift to non-permissive conditions for further 24 h and re-shift to permissive conditions for another day has been documented via microscope.
(3.47 MB TIF)
