# Supplementary figures and images for: Characterization of a Temperature-Sensitive Vertebrate Clathrin Heavy Chain Mutant as a Tool to Study Clathrin-Dependent Events In Vivo
Source: PLoS One. 2010 Aug 6;5(8):e12017. doi: 10.1371/journal.pone.0012017 (PMC2917355; doi:10.1371/journal.pone.0012017)

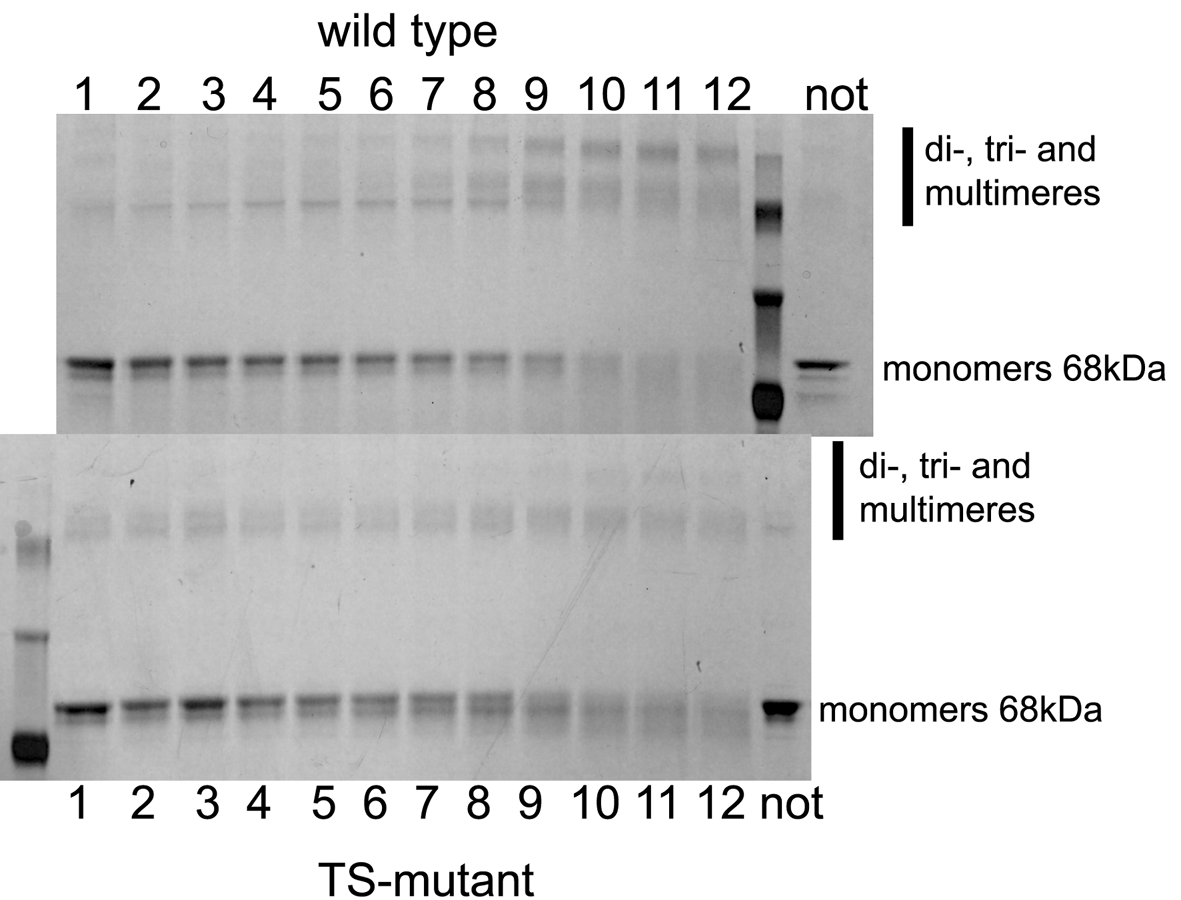

Supplement: Figure S1 — In vitro crosslinking of truncated clathrin triskelions. Purified clathrin heavy chain “hub”-fragments (C-terminus of clathrin heavy chain involved in trimerization) were incubated within a temperature range of 26–47°C for 5 min supplemented with crosslinker. The crosslinking reaction was stopped by adding 4× SDS-PAGE-sample buffer. An equal amount of protein was loaded on a SDS-gradient gel followed by a silver stain. 1 = 25.9°C, 2 = 26.1°C, 3 = 27.2°C, 4 = 28.9°C, 5 = 31.2°C, 6 = 33.8°C, 7 = 36.6°C, 8 = 39.4°C, 9 = 42.0°C, 10 = 44.2°C, 11 = 45.9°C, 12 = 46.8°C, not = no crosslinker added. (3.19 MB TIF) [file pone.0012017.s001.tif]

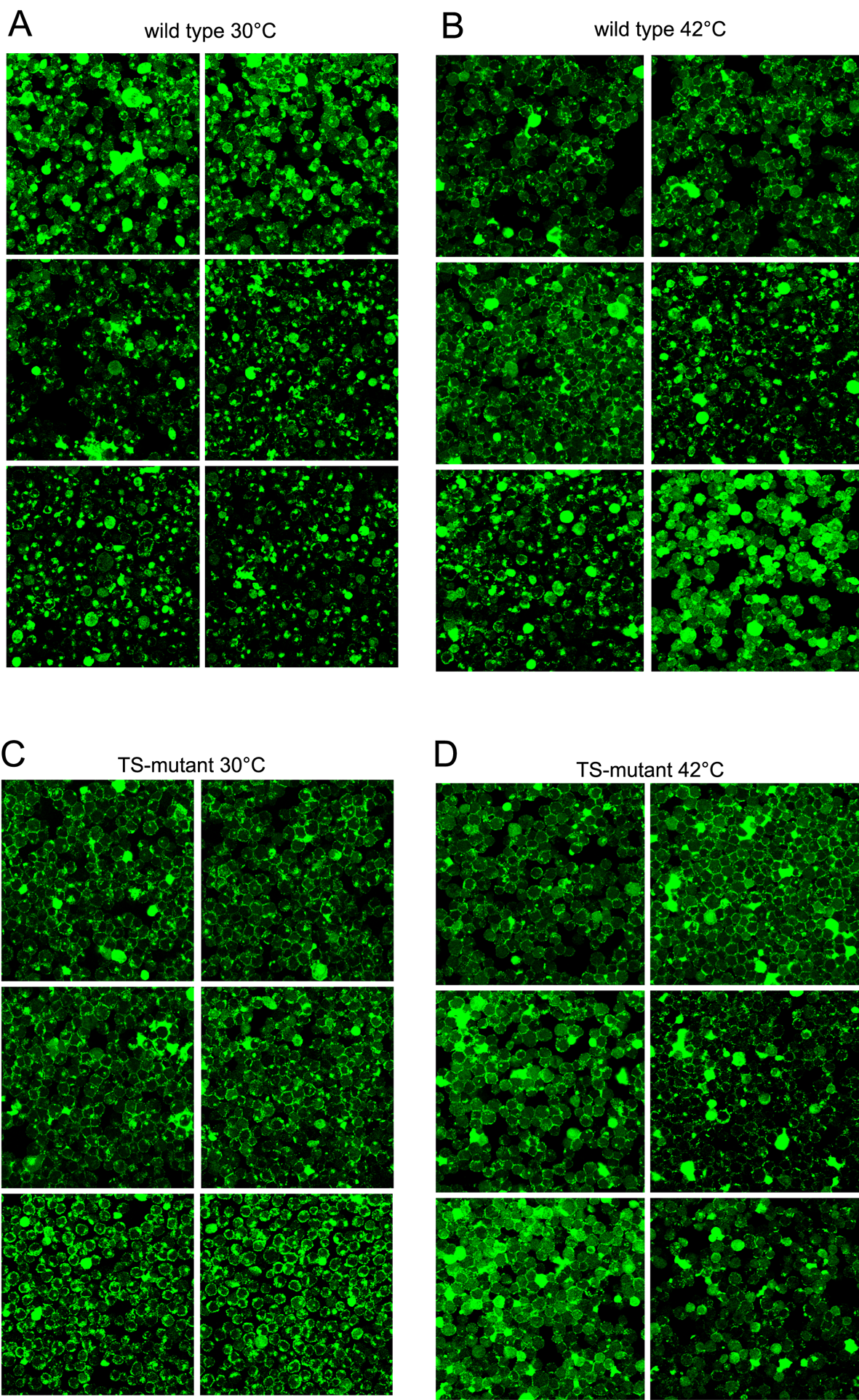

Supplement: Figure S2 — Images to calculate endocytic cells. The endocytosis assay was performed as decribed in figure legend 5. The six individual confocal images 2A–D were used to calculate (cell counter of software ImageJ) the percentage of the cell population which had a prominent intracellular staining, the endocytosed FITC-transferrin. A summary of the results is shown in figure 5B. A = wild type 30°C, B = wild type 42°C, C = TS-mutant 30°C, D = TS-mutant 42°C. (6.82 MB TIF) [file pone.0012017.s002.tif]

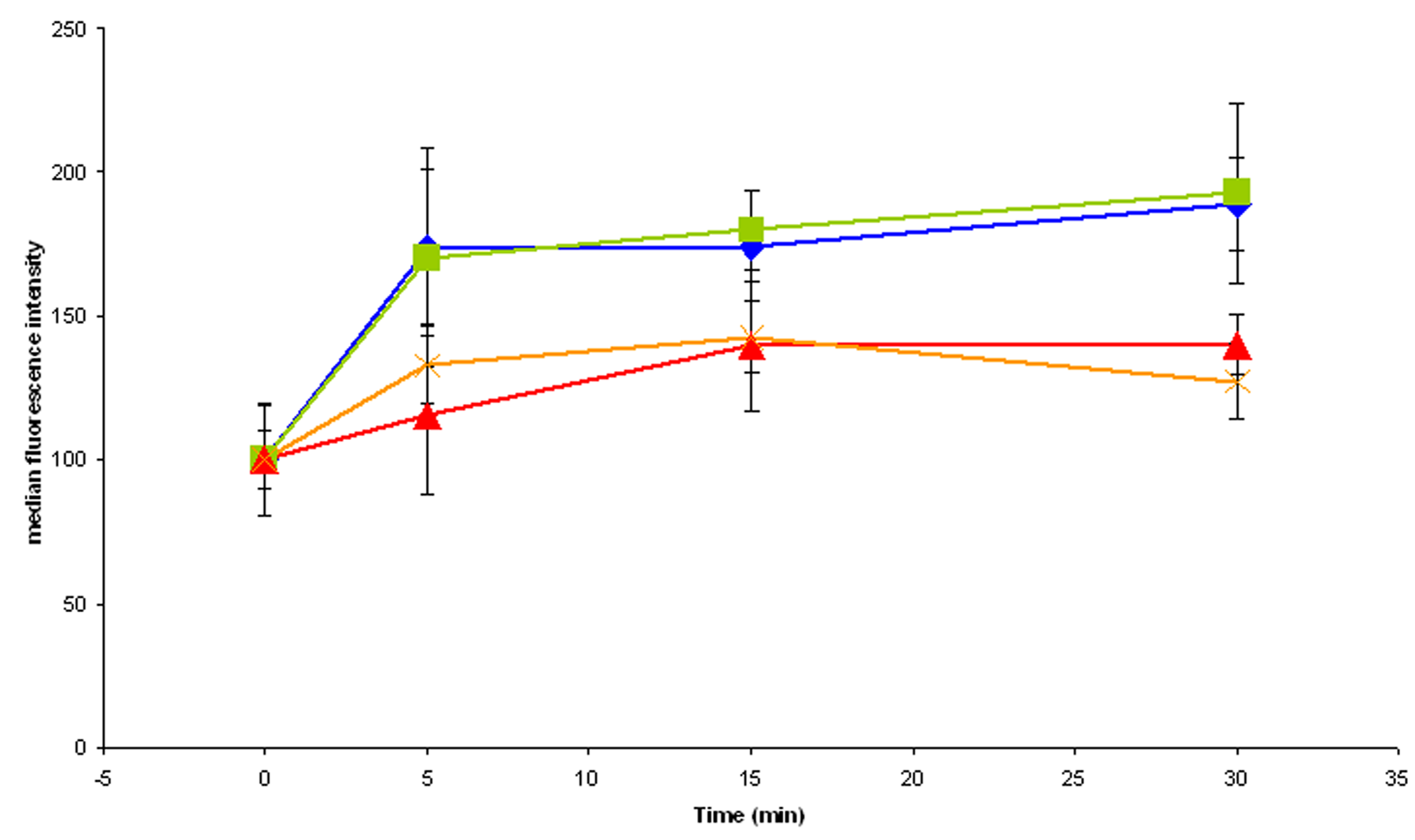

Supplement: Figure S3 — FACS-based quantification of FITC-transferrin endocytosis. Cells were grown for several days at 30°C (TS-cells with doxycycline). For the assay, cells were starved for iron followed by a pre-incubation with FITC-transferrin. Then cells were aliquoted and shifted to either 30°C or 42°C. Over indicated time points cells were analysed via FACScan flow cytometer. Values were normalized against starting value. Blue diamond = wild type 30°C, red triangle = wild type 42°C, green square = TS-mutant 30°C, orange cross = TS-mutant 42°C. (5.78 MB TIF) [file pone.0012017.s003.tif]

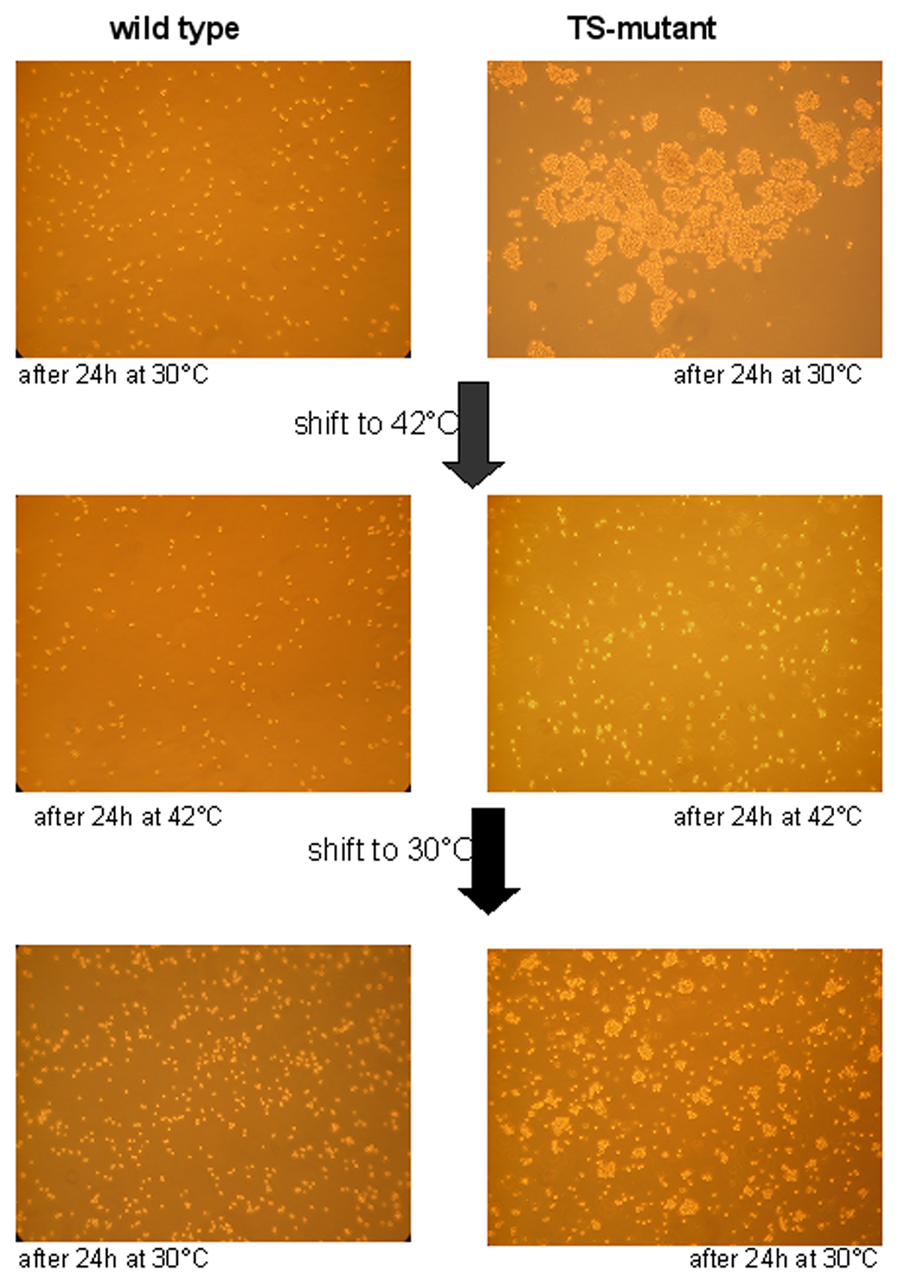

Supplement: Figure S4 — Clustering of TS-mutant cells at the permissive temperature after 24 h of incubation. Clustering of cells grown untouched under permissive conditions for 24 h, followed by shift to non-permissive conditions for further 24 h and re-shift to permissive conditions for another day has been documented via microscope. (3.47 MB TIF) [file pone.0012017.s004.tif]
